# SG-APSIC1093: Engaging inpatients in antibiotic stewardship efforts: The need to enhance knowledge and increase involvement in their antibiotic therapy

**DOI:** 10.1017/ash.2023.5

**Published:** 2023-03-16

**Authors:** Evonne Tay, Angela Chow

**Affiliations:** Singapore; Guo Huiling, Singapore, Tan Tock Seng Hospital, Singapore; Singapore, Tan Tock Seng Hospital, Singapore

## Abstract

**Objectives:** In tertiary-care settings, up to 50% of patients are prescribed at least 1 antibiotic. However, patients are often not proactively provided with information nor involved in shared decisions regarding their antibiotic therapies. Understanding inpatients’ knowledge and the extent of their involvement in antibiotic therapy help reduce inappropriate or unnecessary antibiotic use. **Methods:** A cross-sectional survey was conducted from March to December 2021 in a 1,600-bed, adult, acute-care, tertiary-care hospital. Patients prescribed antibiotics for the past 1 week during their hospital stay were surveyed. Ten questions assessing patients’ knowledge of their antibiotic therapy and 3 questions adapted from the NHS Care Quality Commission Inpatient survey (2013) were included in the survey questionnaire. **Results:** Among the 323 patients surveyed, 88% knew that they had been given antibiotics, and 80% felt that it was important to be informed of the reason, 76% felt that it was important to be informed of side effects, 74% felt that it was important to be informed of duration, and 72% felt that it was important to be informed of dosing frequency. However, only 71% knew the dosing frequency, 54% knew the side effects, 37% knew the duration, and 13% knew the name of the antibiotic agent administered. Of those unaware of the antibiotic name, 59% had indicated their desire to know. Among those aware of their antibiotic therapy, 85% had trust in their doctors but only 42% felt that they always received answers to their questions on antibiotics in an understandable manner from their doctors. Furthermore, 41% felt that they were often or always not given enough time to question their doctors. To raise their awareness on antibiotic use, 73% of respondents felt that having protected time with the doctors to understand more about their antibiotic therapy would be effective. **Conclusions:** Most inpatients lacked knowledge of details of their antibiotic therapy, and fewer than half were involved in it. Allocation of protected time with doctors to understand their antibiotic therapy can be a potentially effective strategy to increase patient engagement to enhance hospital antibiotic stewardship efforts.

